# Supplementary figures and images for: In Silico Identification of lncRNAs Regulating Sperm Motility in the Turkey (Meleagris gallopavo L.)
Source: Int J Mol Sci. 2022 Jul 11;23(14):7642. doi: 10.3390/ijms23147642 (PMC9324027; doi:10.3390/ijms23147642)

A

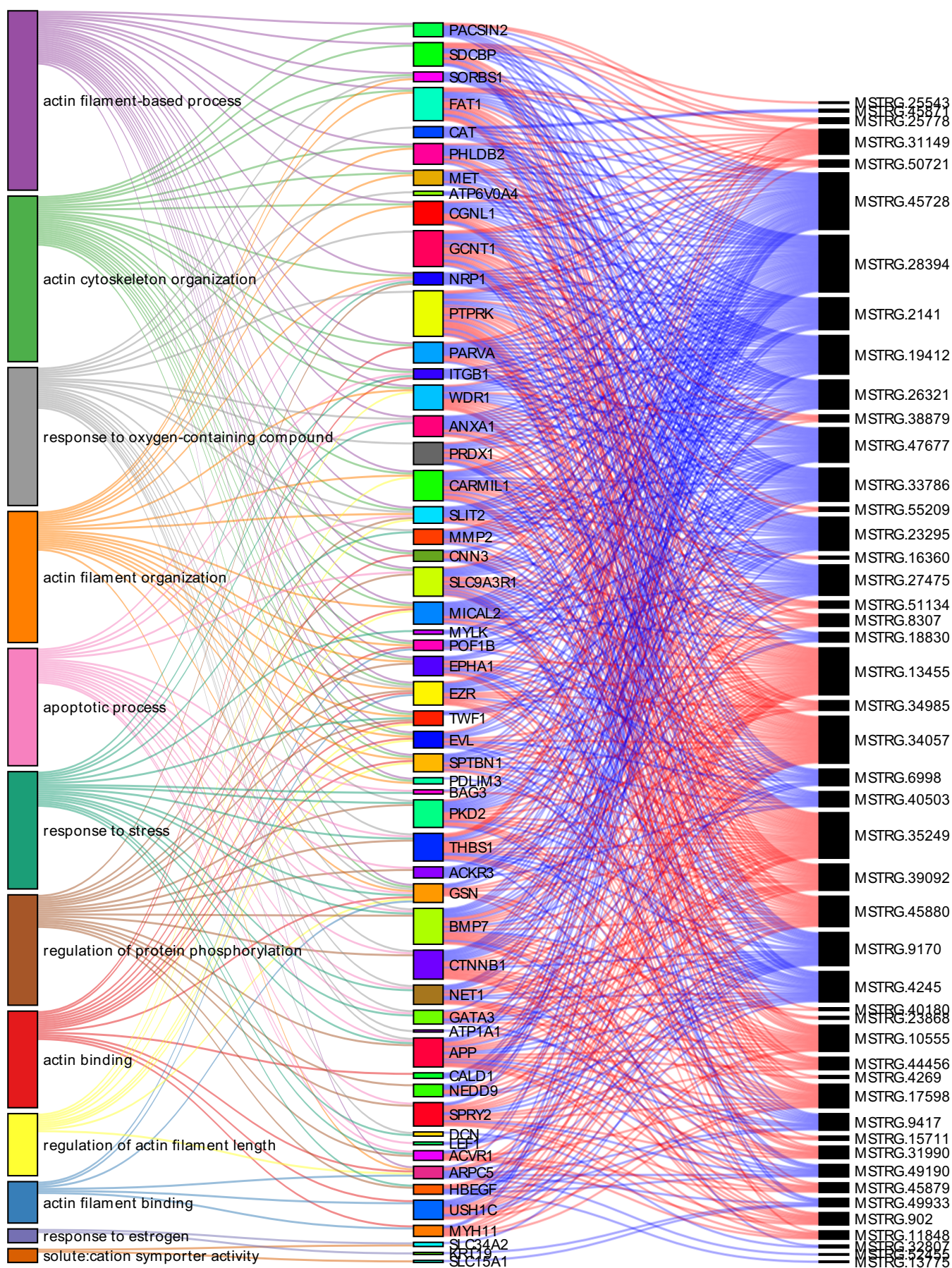

# B

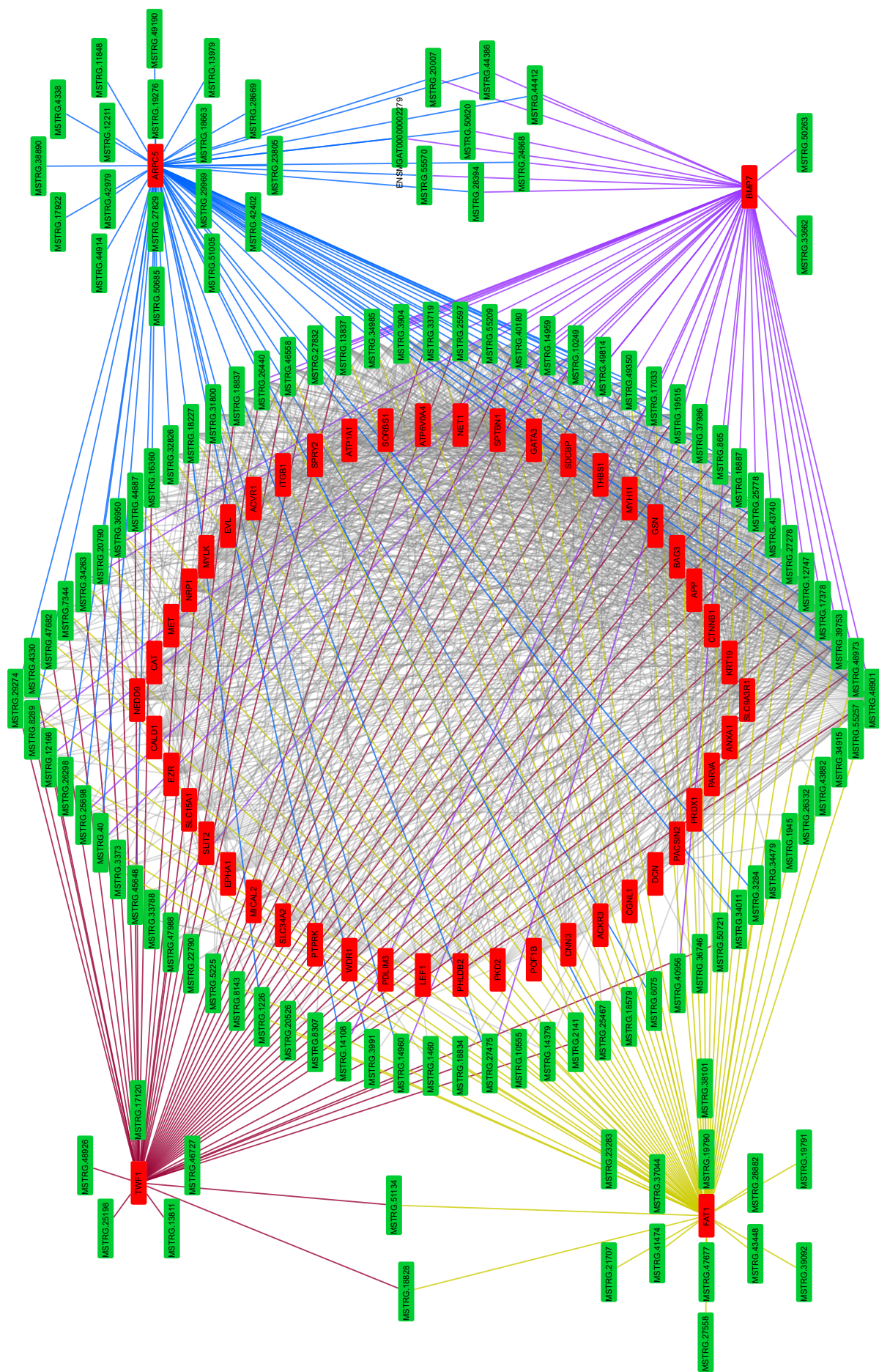

Supplement: Supplementary file 1 [file ijms-23-07642-s001.zip › FigureS1.pdf]

A

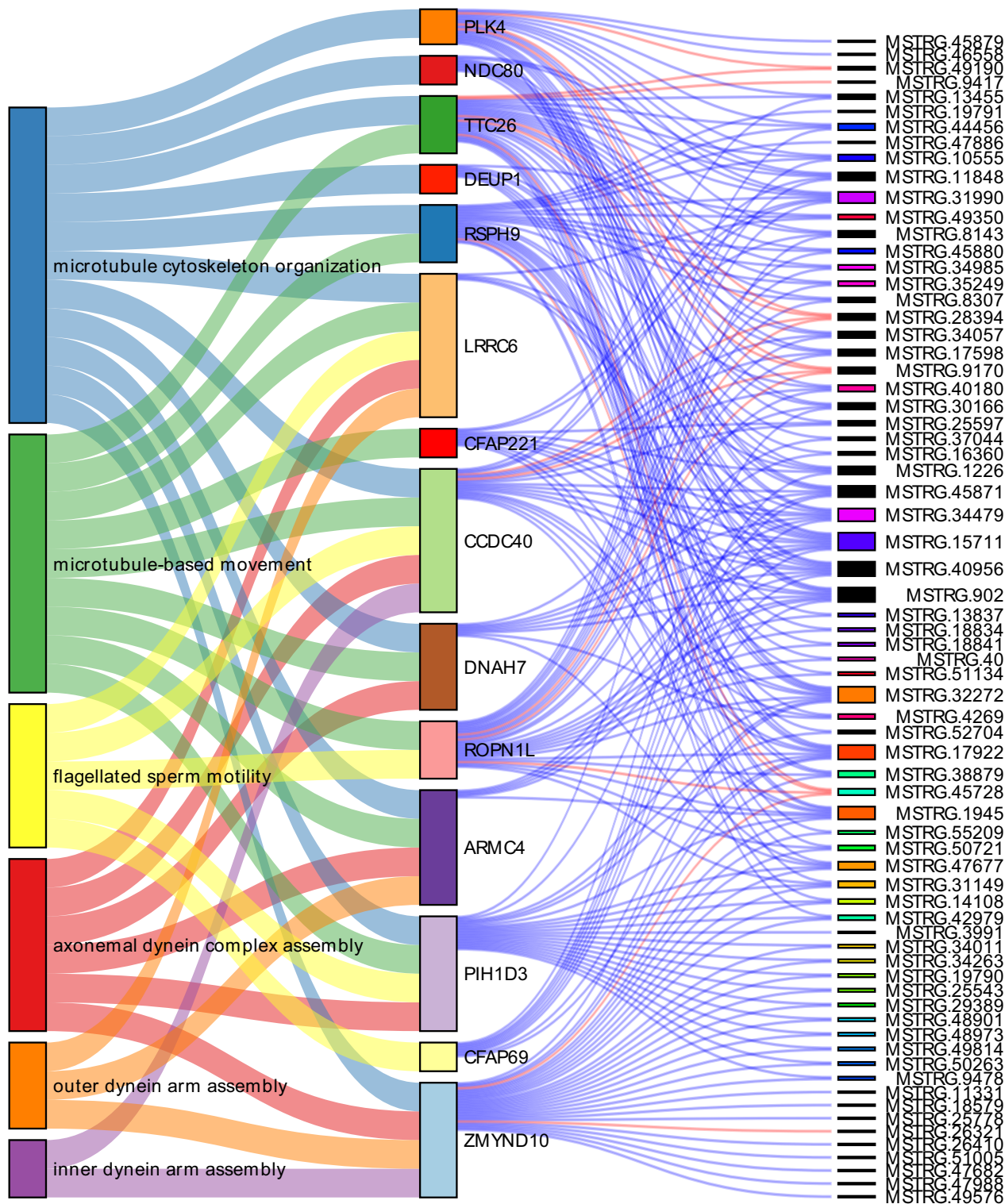

# B

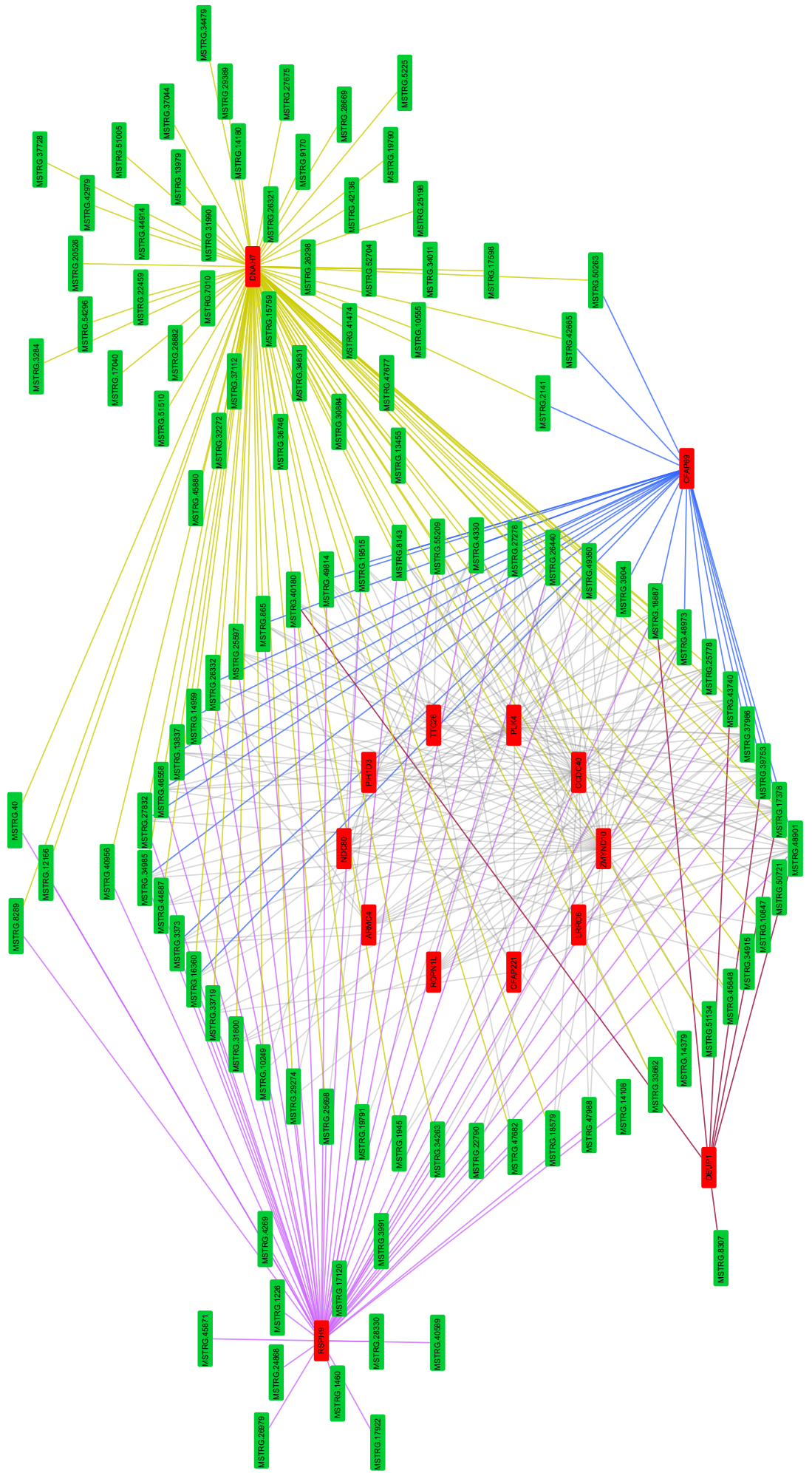

Supplement: Supplementary file 1 [file ijms-23-07642-s001.zip › FigureS2.pdf]
